# Exploring Raoultella planticola: Implications for Pediatric Health

**DOI:** 10.7759/cureus.57262

**Published:** 2024-03-30

**Authors:** Aaron L Bautista, Ramy Wissa, Mariam Fahim

**Affiliations:** 1 Medicine, College of Osteopathic Medicine of the Pacific, Western University of Health Sciences, Pomona, USA; 2 Medicine, University of California, Los Angeles, Los Angeles, USA; 3 Pediatrics, Inscriptions Children's Clinic, Wildomar, USA

**Keywords:** raoultella planticola, e. coli, klebsiella pneumoniae (kp), pediatric infectious disease, vesicoureteral

## Abstract

The case presentation discusses the clinical evaluation and treatment of a two-year-old female exhibiting symptoms such as dysuria, constipation, and foul-smelling urine. Upon evaluation, the patient was found to be co-infected with Raoultella planticola and Escherichia coli. This co-infection poses unique challenges in diagnosis and treatment, as both pathogens may contribute to the manifestation of symptoms. The initial diagnosis of Raoultella planticola is notable, given its relatively rare occurrence and the potential for misdiagnosis. This case study contributes to our understanding of diagnosing and distinguishing symptoms at various stages of the illness, particularly in cases of co-infection. Following an initial urinalysis and urine culture confirming the presence of both pathogens, a 10-day course of antibiotics was prescribed. Subsequent examinations at Rady Children's Hospital-San Diego included kidney and abdomen imaging to rule out underlying issues. The co-infection underscores the importance of thorough diagnostic procedures and tailored treatment approaches. Additionally, it highlights the need for heightened awareness among healthcare providers regarding emerging pathogens and their potential clinical implications.

## Introduction

In recent years, there has been a noticeable increase in *Raoultella planticola* infections among pediatric patients, especially those with congenital anomalies of the kidney and urinary tract (CAKUT), as indicated by a study published in the Translational Pediatrics journal [1]. *R. planticola*, previously known as *Klebsiella planticola* or *Klebsiella trevisanii*, is a gram-negative bacterium commonly found in soil and water sources. While historically considered non-pathogenic to humans, it has been recognized as a cause of rare opportunistic infections, usually seen in immunocompromised and critically ill children admitted in PICU [1,2].

Prior to 2018, only a small number of *R. planticola* infections had been reported, with just seven cases documented in the United States, and pediatric cases were exceptionally rare. However, recent data suggest a growing trend in *R. planticola* infections, particularly bloodstream infections and peritonitis, among pediatric populations, notably those undergoing peritoneal dialysis [2,3].

The emergence of these infections presents significant challenges due to observed antibiotic resistance patterns in *R. planticola *strains. Early identification of these infections is crucial for effective management. Understanding their epidemiology, predisposing factors, and optimal treatment strategies is essential for improving clinical outcomes and implementing preventive measures.

This case report highlights a two-year-old febrile female with a history of recurrent UTIs diagnosed with a urinary tract infection (UTI) caused by *R. planticola*, underscoring the potential pathogenicity of this bacterium in vulnerable pediatric populations. Documenting and studying cases like this are critical for enhancing diagnostic accuracy and ultimately improving patient outcomes. This article aims to contribute to the growing literature on *R. planticola* infections in pediatric patients, thereby assisting clinical practice and public health efforts.

## Case presentation

In July 2023, a two-year-old girl residing in Murrieta, California, presented febrile to a physician with a history of* E. coli *infection, manifesting symptoms of dysuria and malodorous urine persisting for one day. The child had no significant past medical history. According to the mother, the child had spent the previous day seated in a splash pad within their backyard, an area frequented by their pet dogs. Upon physical examination, bilateral erythema was noted in the inner labia minora, with no evidence of discharge or visible trauma. The bilateral erythema at the labia minora is consistent with irritation from UTI. Notably, the patient, who was not yet toilet-trained, had a familial history notable for end-stage renal disease, autism, and behavioral issues. Past speech therapy sessions were discontinued due to perceived inefficacy. A urinalysis conducted during the visit revealed elevated ketone levels, leukocyte esterase, and nitrites. Subsequent clean-catch urine culture identified *Raoultella planticola.* *Raoultella planticola*, displaying typical gram-negative bacilli morphology, exhibited similar characteristics to *Klebsiella pneumoniae* on culture. The identification was confirmed with a certainty percentage of 95% using the MicroScan WalkAway 96 Plus System. Hence, the patient was prescribed a 10-day course of Nitrofurantoin to target *R. planticola*, as their urine report indicated susceptibility to Nitrofurantoin. Co-trimoxazole, previously administered for suspected *E. coli *treatment, was discontinued. Moreover, the patient was referred to the urology department at Rady Children’s Hospital-San Diego for further evaluation, including a voiding cystourethrogram to assess the possibility of recurrent UTIs being attributable to anatomical factors.

In early August 2023, the patient underwent diagnostic examinations at Rady Children’s Hospital-San Diego. These included a kidney and bladder ultrasound, along with an abdominal x-ray, which ruled out the presence of renal calculi. Urodynamics testing yielded negative results. Prophylactic antibiotics were administered before a voiding cystourethrogram to investigate the possibility of vesicoureteral reflux, which ultimately showed no abnormalities. Following the completion of the prescribed antibiotic regimen, a clean-catch urine culture confirmed the resolution of the infection, leading to the patient's discharge.

The anticipated outcome after the two-year-old girl's presentation with febrile urinary tract infection symptoms in July 2023 was a thorough diagnostic assessment followed by targeted treatment and infection resolution. The expectation was for the patient to undergo tests to rule out underlying issues like vesicoureteral reflux, given the symptoms and family medical history. Treatment with prescribed antibiotics, Nitrofurantoin and Co-trimoxazole, was expected to clear the identified pathogens. However, the actual outcome exceeded expectations. Tests at Rady Children’s Hospital-San Diego showed normal kidney and bladder ultrasound results, and no significant issues on abdominal X-ray. The repeat urine culture confirmed infection resolution after completing the antibiotic course, leading to the patient's discharge without complications, achieving a positive result.

## Discussion

Differential diagnoses

When considering a *Raoultella planticola* infection, several differential diagnoses should be taken into account to ensure comprehensive evaluation and appropriate management. Firstly, urinary tract infections caused by other bacterial pathogens such as *Escherichia coli*, *Klebsiella pneumoniae*, and *Proteus mirabilis* should be considered, given their common occurrence and similar clinical presentations [1]. Additionally, in pediatric cases presenting with dysuria and foul-smelling urine, non-infectious etiologies such as urinary tract abnormalities, anatomical anomalies, and functional disorders like voiding dysfunction or urinary retention should be explored. Other infectious causes of urinary symptoms, including viral and fungal pathogens, must also be considered, especially in immunocompromised individuals. Furthermore, given the patient's history of *E. coli* infection and exposure to animals, zoonotic infections transmitted through contact with animals or contaminated environments should be evaluated. These may include bacterial pathogens such as *Campylobacter spp.*, *Salmonella spp.*, or *Yersinia spp.* and parasitic infections like *Giardia lamblia* or *Cryptosporidium spp*. Overall, a thorough assessment considering both infectious and non-infectious differential diagnoses is crucial for accurate diagnosis and optimal management of *Raoultella planticola* infection.

The condition

*Raoultella planticola*, a gram-negative bacterium belonging to the *Enterobacteriaceae* family, is recognized as an opportunistic pathogen that predominantly affects immunocompromised individuals and those with preexisting medical conditions [2, 3]. Although not commonly associated with urinary tract infections (UTIs), sporadic cases have been documented, emphasizing the importance of vigilance in detecting and reporting such infections for public health monitoring purposes. Documenting *Raoultella planticola* infections aids in assessing prevalence, tracking resistance patterns, and identifying potential outbreaks, thereby offering valuable insights into healthcare trends.

By 2018, documented cases of severe human infections caused by *Raoultella planticola* numbered 32, spanning from 1984 to 2018. Among these cases, six were attributed to UTIs, with two occurring in pediatric patients [4]. Our report contributes to this body of knowledge by documenting a third instance of a pediatric UTI caused by *Raoultella planticola*.

While *Raoultella planticola* is typically found in soil, water, and human sputum, its association with UTIs is uncommon [4]. In pediatric cases, *Escherichia coli* is the predominant causative agent, responsible for approximately 80 percent of UTIs in children. Additionally, other gram-negative pathogens, including *Klebsiella*, *Proteus*, *Enterobacter*, and *Citrobacter*, are commonly implicated.

Although infections caused by *Raoultella planticola* are infrequent, reported cases have shown a recent increase [4, 5]. Initially considered a subspecies of *Klebsiella pneumoniae*, *Raoultella planticola *was reclassified as a distinct genus in 2001 while remaining within the *Enterobacteriaceae* family [6]. Similar to other *Enterobacteriaceae* bacteria such as *Klebsiella*, *Raoultella planticola* may exhibit carbapenemase activity, rendering it resistant to carbapenem antibiotics, which are crucial as a last resort for treating multidrug-resistant bacterial infections [7].

To date, only one documented case exists in the literature, reported by Tseng et al., detailing a pneumonia case caused by a carbapenem-resistant strain of *Raoultella planticola* isolated from sputum culture [7]. Resistance to carbapenems in *Raoultella planticola* is attributed to the presence of carbapenemase genes, often located on plasmids, facilitating horizontal gene transfer among various bacteria.

The emergence of carbapenem-resistant *Raoultella planticola *underscores the importance of stringent infection control measures, judicious antibiotic use, and ongoing surveillance to combat antibiotic resistance in healthcare settings and the community. Additionally, research into novel treatment strategies and efforts to address antibiotic resistance remain paramount in infectious disease management.

Treatment

Management of *Raoultella planticola* infections usually requires antibiotic therapy tailored to the susceptibility profile of the specific strain. Considering the increasing resistance of certain strains to carbapenem antibiotics, empirical therapy may not be recommended until a sensitivity report becomes available. However, definitive therapy should be guided by culture and susceptibility testing results to ensure optimal efficacy and minimize the risk of further antimicrobial resistance. In cases of carbapenem-resistant strains, alternative antibiotic options, such as polymyxins or tigecycline, may be considered, although these agents often carry greater risks of toxicity and limited clinical data in pediatric populations [7]. Alongside antibiotic therapy, supportive measures such as hydration and symptom management may be necessary, particularly in severe or systemic infections. Close monitoring of clinical response and repeated cultures to assess for treatment efficacy and the emergence of resistance are essential components of managing *Raoultella planticola *infections.

Lessons for the clinician

Clinicians should maintain a high index of suspicion for uncommon pathogens, particularly in patients with predisposing factors such as immunocompromised states or unusual environmental exposures.

While *Raoultella planticola *infections are rare, their incidence appears to be increasing, underscoring the importance of vigilance in microbiological surveillance and reporting. Predisposing factors such as immunocompromised states, recent hospitalizations, and invasive medical procedures should also be considered in suspected cases.

The emergence of carbapenem-resistant strains poses significant therapeutic challenges, necessitating judicious antibiotic selection and consideration of alternative treatment options based on susceptibility testing results.

Effective management of *Raoultella planticola* infections requires a multidisciplinary approach, involving collaboration between clinicians, microbiologists, and infection control specialists to optimize patient outcomes and mitigate the risk of antimicrobial resistance.

## Conclusions

In conclusion, this case report highlights the significance of vigilant monitoring, particularly in pediatric patients with recurrent UTIs. The clinical presentation of an afebrile two-year-old girl with dysuria and malodorous urine underscores the importance of considering subtle symptoms in diagnosing and managing urinary tract infections in children. Given the potential for complications and recurrence in this population, heightened vigilance and thorough evaluation are necessary to ensure optimal care. As such, collaborative efforts among healthcare professionals, researchers, and public health authorities are crucial in enhancing surveillance and reporting mechanisms, thereby strengthening disease control and prevention strategies. This collective approach is vital not only for managing individual cases effectively but also for safeguarding global health security in the face of emerging infectious diseases.
